# Cardiopulmonary Arrest During Pregnancy: A Review Article

**DOI:** 10.7759/cureus.35219

**Published:** 2023-02-20

**Authors:** Sujeet J Pawar, Vaibhav P Anjankar, Ashish Anjankar, Mohammad Adnan

**Affiliations:** 1 Anatomy, Jawaharlal Nehru Medical College, Datta Meghe Institute of Higher Education and Research, Wardha, IND; 2 Biochemistry, Jawaharlal Nehru Medical College, Datta Meghe Institute of Higher Education and Research, Wardha, IND

**Keywords:** haemorrhage, maternal mortality, foetal survival, pulmonary embolism, cardiac arrest

## Abstract

Massive pulmonary embolism (PE) is an uncommon but severe complication of pregnancy or during the first few weeks after giving birth. Our intention was to thoroughly analyze the information available to its management methods. Significant bleeding of mother survival and early deliveries in fetal survivals were having hemorrhage and were having key outcomes. We found 127 severe PE cases that have had at least one form of treatment (at least 83% big; 23% with cardiac arrest). The 83 women who received thrombolysis had a 94% (95%) survival rate. Cardiac arrest in pregnancy is uncommon, although maintaining current competency can be challenging. While maternal mortality rates have decreased globally over the past 25 years, they have increased in the United States. The intricacy of the maternal mortality issue is a result of a number of clinical and socioeconomic problems such as unequal healthcare access, racial and ethnic disparities, maternal comorbidities, and bias in epidemiologic ascertainment. The importance of doctors being prepared to react to a potential maternal cardiac arrest in any situation where they are providing treatment for pregnant women is highlighted by the rise in maternal mortality. For the treatment of maternal cardiac arrest, an interdisciplinary team with expertise in both the maternal resuscitation procedure and the physiological changes that take place during pregnancy is necessary. Additionally offered are basic and advanced cardiac life support protocols. Techniques to remove obstacles like aortocaval compression that could impair the effectiveness of resuscitation should be used.

## Introduction and background

Cardiovascular arrest during pregnancy is one of the most challenging clinical scenarios. Although resuscitating a pregnant woman shares many characteristics with resuscitating an adult, there are several specific differences. The fact that there are two patients -- the mother and the fetus is the most noticeable distinction [1]. Due to inadequate pre-hospital care, a lack of skilled staff, and incorrect interhospital transfers, first resuscitation within the "golden hour" frequently goes unperformed. But when a pregnant woman experiences polytrauma, the outcome is frequently disastrous [2]. In India, there are around one in 12 hospitalizations for childbirth. Although the underlying etiology heavily influences maternal outcomes, the prognosis is generally favorable, with up to 58% of patients surviving to the hospital discharge. Maternal mortality has increased over the past 20 years and that for the treatment of maternal cardiac arrest, an interdisciplinary team with expertise in both the maternal resuscitation procedure and the physiological changes that take place during pregnancy is necessary. Additionally offered are basic and advanced cardiac life support protocols [3]. Maternal collapse occurs in one in every 36,000 pregnancies. Hypertensive, diabetics, and elderly primigravida are more likely to suffer from it. Medical problems that are present with many pregnancies can greatly increase the circulatory strain on the mother and raise the risk of a maternal collapse. When maternal collapse unfortunately happens, resuscitative hysterotomy, often called a perimortem cesarean section, is a procedure that is frequently carried out during the second and third trimesters of pregnancy. Immediate hysterotomy may be necessary if the lady does not react to first cardiopulmonary resuscitation (CPR) and manual uterine displacement [4]. Anesthesia problems are a substantial cause of maternal cardiac arrest in affluent countries. Bleeding and infections are also frequent. Recent in-depth research understrike the need for fractionated care for pregnant women with cardiac and maybe neurological disorders. Thromboembolic episodes are more frequent than previously believed, but they are still rare, according to pathology research. High death rates go in hand with them. As opposed to the majority of cardiac arrest populations, the presenting rhythms of cardiac arrest underscore the need for more in-depth research into the causes of and approaches to treating these situations [5].

Although adult CPR was developed in the 1960s, it took another 30 years for pregnant women to receive this treatment. Additionally, it did not take up much room in the clinical practice recommendations. The intricacies of maternal CPR are now more clearly understood and are covered in several portions of the recommendations. Pregnancy-related physiological alterations to the cardiovascular system must be taken into consideration for CPR on pregnant women to be effective. Pregnancy causes an increase in cardiac output, blood volume, respiration, and oxygen intake. Additionally, particularly while in the dorsal decubitus position, the gravid uterus compresses the abdomen and iliac arteries, which decreases blood pressure and cardiac output. To take these physiological alterations into account, the standard first therapy for cardiorespiratory arrest must be modified [6]. Cardiovascular and circulatory failure can appear during pregnancy and in the first few weeks following delivery as a result of obstetric emergency as well as a side effect of underlying cardiac or vascular abnormalities. A cardiac arrest is still a rare event, thus it must be prepared for by training caregivers and having resuscitation equipment available. Negative outcomes for the mother or the fetus are more frequent in pregnant women with underlying cardiovascular vitae (CV) problems. Modern resuscitation methods have been administered to this population. The majority of the causes of the subgroup of maternal cardiac arrests that occurred in the delivery room or after delivery (estimated frequency) were acute myocardial infarction, venous embolism, and amniotic fluid embolism [7].

## Review

Methodology 

**Figure 1:**
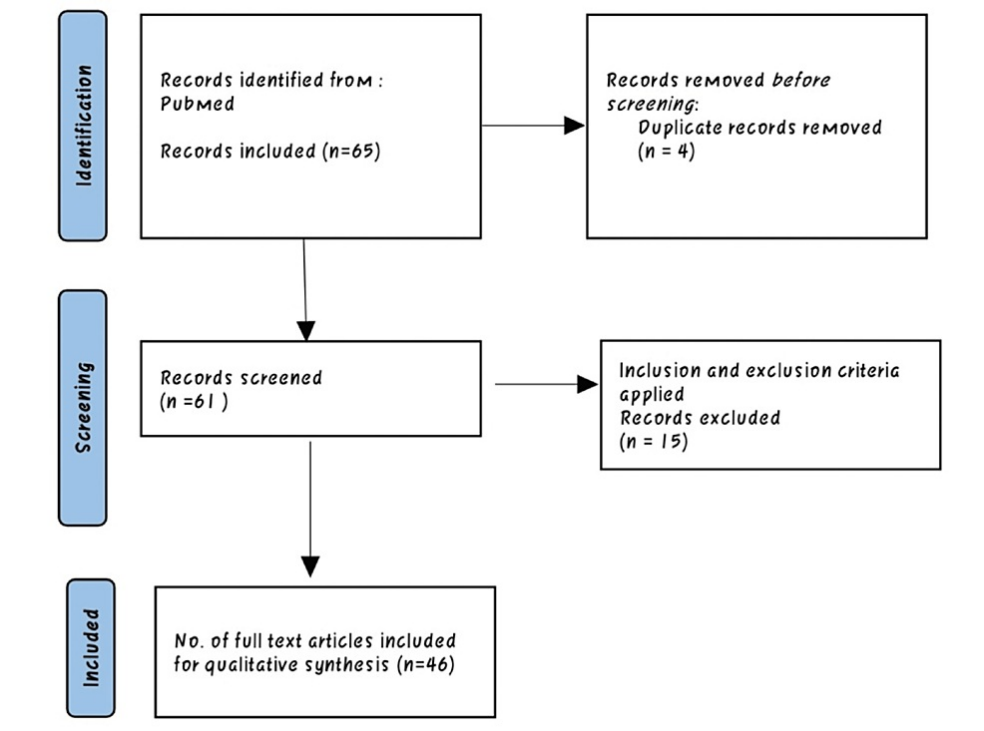
Inclusion exclusion criteria.

We undertook a systematic search through PubMed and CENTRAL in November 2020 using keywords such as "tuberculosis" and " quality of life" ((tuberculosis [Title/ Abstract]) OR (TB (Title/ Abstract.])) OR (koch*[Title/Abstract)) OR ("tuberculosis" [MeSH Terms]) AND ("quality of life" [Title/ Abstract]) OR (QoL [Title/ Abstract))) OR ("quality of life" [MeSH Terms). We additionally searched for key references from bibliographies of the relevant studies. The search was updated in February 2022. One reviewer independently monitored the retrieved studies against the inclusion criteria, in the beginning, based on title and abstract and then on full texts. Another reviewer also reviewed approximately 20% of these studies to validate the inclusion of studies (Figure 1).

Around 2.73 cardiac arrests occurred for every 10,000 spinal anesthesia, which is consistent with previous research suggesting that cardiac arrest during spinal anesthesia is uncommon. Perioperative cardiac arrest occurred in 4.3 out of every 10,000 patients and occurred in 1.5 out of every 10,000 patients who underwent regional anesthesia. There are 6.4 cardiac arrests for every 10,000 spinal anesthetics, with a 77% survival rate. Some 11 cardiac arrests were recorded out of 101,769 narcotic, indicating a cardiac arrest frequency of six per 10,000 neuraxial anesthetics, and a 20% survival rate. For every 10,000 spinal narcotic, there were 2.9 cardiac arrests, according to Kopp et al. In our study, intraoperative cardiac arrest was more likely to occur in patients with shorter statures, higher American Society of Anesthesiologists (ASA) physical status, lengthier operations, and surgeon-performed spinal anesthesia. Multivariate analysis revealed that patients with shorter stature had statistically significant probabilities of going into cardiac arrest during surgery [odds ratios of 0.944 (95% CI: 0.938-0.951), 1.003 (95% CI: 1.001-1.005), and 23.508 (95% CI: 6.11-90.415) for surgeons administering spinal anesthesia). More than any other risk factor, the patient's height is more likely a proxy for cardiac arrest. For instance, females accounted for nine out of 11 cardiac arrest incidents. We also noted a connection between longer surgical procedures and cardiac arrest [8]. Even though pregnancy and delivery are mostly risk-free for both mother and child in the United States, major maternal problems, such as cardiac arrest, can develop throughout the prenatal, intrapartum, and postpartum phases. In the course of their careers, clinical obstetricians can anticipate running into this condition. To help emergency medicine and critical care physicians revive the patient, the obstetrician must be informed of the unique circumstances surrounding the resuscitation of the gravid lady. The obstetrician is responsible for comprehending the decision-making process that results in a perimortem cesarean and for carrying out the cesarean delivery [9]. The frequency of heart illness has been rising since 1991, according to the Confidential Enquiries into Maternal and Child Health report, and it causes 2.27 deaths per 100,000 pregnancies. Between 1% and 4% of pregnancies are complicated by acquired heart disease, which also significantly increases morbidity and mortality. The most common cause of mitral stenosis in the developing world (and among immigrants residing in Western nations) is rheumatic fever. Congenital cardiac disease is a common condition in the United States. All of the diseases previously mentioned, as well as other conditions, are potential causes of cardiac arrest during pregnancy. Obtaining intravenous (IV) access above the diaphragm, giving 100% oxygen, and alleviating inferior vena cava compression are all parts of the initial therapy. There are numerous ways to relieve aortocaval compression [10]. CARPREG II risk scores. Atrioventricular valve regurgitation with subpulmona was designated by tricuspid valve regurgitation (TR), while systemic and/or subaortic atrioventricular regurgitation was denoted by mitral valve regurgitation (MR) (risk categories: 0-1) [11]. Management of pregnancy: A non-triggering anesthetic (local, nerve block, epidural, spinal anesthesia, or a full IV general anesthetic) should be used if a pregnant woman with a Master of Health Science (MHS) needs non-emergent surgery. For labor and delivery, continuous epidural analgesia comes highly recommended. If a woman needs a cesarean delivery but does not already have an epidural catheter in place is advised to use neuraxial anesthesia (spinal, epidural, or spinal-epidural combined) (if not otherwise contraindicated). If a general anesthetic is necessary, it should be administered using a full IV anesthetic strategy on an anesthesia machine that has been set up for a patient who is MH-susceptible [12].

Pregnancy in arrhythmogenic cardiomyopathy 

At 32 weeks of gestation, cardiac output can increase by as much as 80% from the starting point. By the conclusion of the first trimester, three-quarters of this increase has been produced, thanks to a rise in heart rate afterwards and an increase in stroke volume during the first half of pregnancy. Systolic right ventricle (RV) and left ventricle (LV) performance are unaltered by the 30% increase in ventricular diameter. Additionally, preload blood volume increases by 40% during pregnancy compared to baseline, and systemic vascular resistance falls. During pregnancy, these physiological adaptations cause biventricular dilatation and eccentric LV hypertrophy, which afterwards go away within six months after delivery. The increase in cardiac output that happens during childbirth is brought on by uterine contractions, discomfort, and fear. Due to autotransfusion, cardiac output jumps by 80% right away after delivery. It then rapidly declines within 10 min and returns to pre-labor levels within 24 h [13].

Discussion

The perinatal prognosis was good despite the significant maternal mortality rate in this case series (6.38%) [14]. Maternal death is still a significant issue, despite developments in the field of pulmonary atrial hypertension (PAH) and evolving treatment modalities over the years, as we found in our thorough systematic investigation of pregnancy-related outcomes in women with PAH. Maternal mortality was 11% in the first trimester and 12% in the second trimester. The first week after delivery was the period with the highest risk, and it was also when 61% of maternal deaths occurred. There were 4% pregnancy-related deaths. Of the live births, 58% of the newborns were preterm and frequently had low birth weights. In the paragraphs that follow, we discuss the clinical care implications of our findings and contrast them with earlier reviews [15]. Catecholaminergic polymorphic ventricular tachycardia is characterized by episodic syncope that happens during vigorous exercise or extreme emotion. Catecholaminergic polymorphic ventricular tachycardia (CPVT). These episodes are initiated by the onset of ventricular tachycardia (bidirectional or polymorphic). It is possible for these arrhythmias to spontaneously end when they do. Ventricular tachycardia in other circumstances may evolve into ventricular fibrillation and cause fast death if cardiopulmonary resuscitation is not immediately accessible. However, symptoms have been observed to occur as late as the fourth decade of life. The typical age at which symptoms (frequently a syncopal episode) first appear is seven to 12 years old. CPVT is a condition in which up to 80% of people suffer one or more syncopal periods and about 30% of people with the condition experience at least one cardiac arrest [16]. On both the mother's and the child's health. A 1.6 (95% CI: 1.3-2.0) higher likelihood of an obstetric near miss was seen in women with severe mental illness (SMI). This was particularly the case for women who had used mental health services for a longer period, suggesting that more severe or persistent mental illness may increase overall rates [17]. Sudden cardiac arrest (SCA) is more common in males than in women, and it is linked to several illnesses in both sexes. Compared to less than 50% in women, coronary heart disease accounts for 80% of deaths in males. Women who survive an SCA are more likely to have physically normal hearts and appear to be healthy, as well as inherited causes such as long QT syndrome (LQTS). Exact studies have shown that women who experience high levels of stress are protected from sudden cardiac death. Women's gender-specific variations in adrenergic transmission during times of stress may act as a built-in defence mechanism (Table 1) [18].

**Table 1:** Main causes of cardiac arrest in pregnancy.

Obstetric causes	Non-obstetric causes
Hemorrhage	Pulmonary embolism
Pregnancy-induced hypertension	Sepsis
Peripartum cardiomyopathy	Stroke
Anesthesia complications	Myocardial infarction
Amniotic fluid embolism	Trauma

According to recommendations for the care of if there is no response to carefully administered CPR and properly regulated uterine displacement during a pregnancy-related cardiac arrest, the choice, to deliver the infant should be considered. When the uterus can be felt above the umbilicus, which can happen as early as 16 weeks in multiple pregnancies and after 20 weeks of Gesta resuscitative hysterotomy is necessary to make mother resuscitation possible. Preeclampsia and prenatal hypertension are two hypertensive disorders of pregnancy that complicate around 8% of pregnancies globally. These disorders have long-term cardiovascular effects in addition to dramatically raising the risk of morbidity and mortality during pregnancy [19]. The key priorities should be providing high-quality CPR to pregnant women experiencing cardiac arrest and reducing aortocaval compression with left-lateral uterus displacement, according to the 2020 American Heart Association (AHA) Guidelines. Pregnancy physiology, extrapolations from non-arrest pregnancy states, and research employing non-randomized simulations all largely corroborate this guidance. The recommendation is difficult to understand because it was based on inconsistent data, including non-randomized simulation-based studies done 30-40 years ago [20]. Torsade de pointe (TdP) ventricular tachycardia is the most common tachyarrhythmia associated with the long QT syndrome (LQTS), which is defined by QT prolongation and aberrant T-waves on the electrocardiogram (ECG) TdP. The most typical symptom of people with LQTS is a syncopal event, which TdP typically causes by self-terminating. These cardiac episodes commonly happen without notice while exercising or under mental stress, but less frequently while sleeping. TdP can occasionally progress to ventricular fibrillation, which results in abrupt death or abortive cardiac arrest (if the patient is defibrillated). One to several syncopal events is typically present in 50% of untreated people with a pathogenic mutation in one of the genes linked to LQT [21]. The majority of congenital cardiac abnormalities may now be reliably diagnosed during pregnancy because of advances in high-resolution ultrasound technology. In the United States, the second trimester has long been used to screen the four-chamber segment. Some 60% of serious cardiac issues can only be found by looking at the heart during a four-chamber section, and even then, it takes later echocardiography throughout pregnancy to spot an abnormal four-chamber section. This approach for cardiac screening during the first trimester of pregnancy has just recently become available [22].

When compared to healthy controls, pregnant women with gestational diabetes mellitus (GDM) showed considerably worse cardiac function. Conventional and speckle-tracking echocardiography tests on women with GDM and controls showed significantly increased LV-RWT and decreased left ventricular global longitudinal strain (LV-GLS), LV endocardial, and epicardial GLS. These subclinical alterations in GDM women who looked to be carrying a healthy term child indicate a markedly maladaptive cardiovascular response [23].

Only in small-for-gestational-age and preterm infants was a difference in maternal angiogenic factors associated with a higher juvenile left ventricular mass, as demonstrated by lower maternal first and second-trimester. Placental growth factor (PlGF) concentrations and increased salt-1 concentrations [24]. We examined the plausibility of TTM for treating post-cardiac arrest brain injury in pregnant women as well as the efficacy of tPA for treating substantial pulmonary embolism (PE) that causes cardiac arrest [25]. Patients with neurocritical illnesses frequently experience seizures and enter status epilepticus (SE). Pregnancy-related factors comprise the eclamptic spectrum, medication non-compliance, drug metabolism problems, acute symptomatic status epilepticus (after an abrupt brain trauma), and spontaneous new-onset refractory status epilepticus (NORSE) [26]. Twins have a greatly increased risk of preeclampsia. Pregnancies compared to singleton pregnancies. Its issues cause the illness to deteriorate and appear more quickly. On the other hand, moms with twins are usually either totally excluded from the study's singleton group or excluded altogether [27]. Pregnancy-related heart disease increases the risk of serious, perhaps deadly cardiac and obstetric issues. Most of these cardiac events are preventable. It is imperative to implement severe maternal cardiac issues prevention methods in this population of women who are at high risk [28].

Some limitations exist in our research. Since Medicaid claims data is a claims database rather than a research database, which has limitations, it cannot be utilized to report rates of health outcomes. The US Medicaid Analytic extract, a database that tracks the amount of money spent on healthcare nationally, has pre-eclampsia analysis. The examination of hospital twenty two revealed that pre-eclampsia had a positive predictive value of 67% (95% CI 54%-77%). The hospital should create policies for a patient who is pregnant and going to be arrested. This makes it possible to activate the aforementioned groups quickly and simply [29]. In women with complete atrioventricular block (CAVB), all cardiac events happened after delivery, and postpartum cardiac events were predicted by a family history of CAVB and perinatal ventricular pause [30]. Recent years have seen an increase in the prevalence of PE, mostly due to modern risk factors such as chronic disease, obesity, and cancer [31]. In a recent data set of pregnant women during a hospital stay for birth in the United States, pulmonary hypertension (PH) was linked to an increase in major adverse cardiovascular events (MACE). With a particularly significant probability among those who also had concomitant cardiomyopathy [32]. Ectopic pregnancies caused by cesarean delivery are more likely to occur in women who have had one before. For the mother, these pregnancies can be potentially fatal consequences [33].

## Conclusions

Heart failure is an interesting complexity of pregnancy that all obstetricians ought to be ready for, particularly given the rising number of ladies with high gamble pregnancies. Early acknowledgment of capture and quick commencement of maternal revival can fundamentally work on maternal and fetal results. All suppliers who work intimately with pregnant patients ought to know about the most recent 2015 AHA rules for the board of heart failure in pregnancy.
